# Interrogating the Light-Induced Charging Mechanism in Li-Ion Batteries Using *Operando* Optical Microscopy

**DOI:** 10.1021/acs.nanolett.3c01148

**Published:** 2023-08-08

**Authors:** Raj Pandya, Angus Mathieson, Buddha Deka Boruah, Hilton B. de Aguiar, Michael de Volder

**Affiliations:** † Laboratoire Kastler Brossel, ENS-Université PSL, CNRS, Sorbonne Université, Collège de France, 24 rue Lhomond, 75005 Paris, France; ‡ Department of Physics, Cavendish Laboratory, University of Cambridge, JJ Thomson Avenue, Cambridge CB3 0HE, United Kingdom; § Department of Engineering, University of Cambridge, Cambridge CB3 0FS, U.K.; ∥ Institute for Materials Discovery, University College London, London WC1E 7JE, U.K.

**Keywords:** optical microscopy, photobatteries, vanadium oxide, *operando* imaging

## Abstract

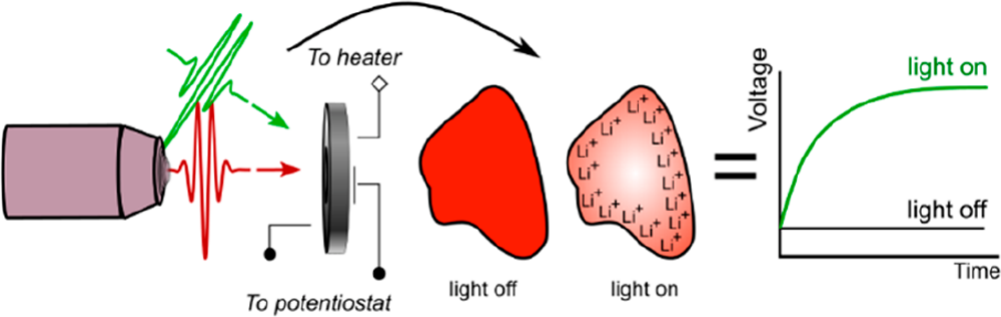

Photobatteries, batteries with a light-sensitive electrode, have recently been proposed as a way of simultaneously capturing and storing solar energy in a single device. Despite reports of photocharging with multiple different electrode materials, the overall mechanism of operation remains poorly understood. Here, we use *operando* optical reflection microscopy to investigate light-induced charging in Li*_x_*V_2_O_5_ electrodes. We image the electrode, at the single-particle level, under three conditions: (a) with a closed circuit and light but no electronic power source (photocharging), (b) during galvanostatic cycling with light (photoenhanced), and (c) with heat but no light (thermal). We demonstrate that light can indeed drive lithiation changes in Li_*x*_V_2_O_5_ while maintaining charge neutrality, possibly via a combination of faradaic and nonfaradaic effects taking place in individual particles. Our results provide an addition to the photobattery mechanistic model highlighting that both intercalation-based charging and lithium concentration polarization effects contribute to the increased photocharging capacity.

Low-power consumption devices such as wireless sensor networks are playing an increasingly important role in the 21st century.^1−4^ With >1300 W m^–2^ of solar radiation reaching the surface of the earth annually,^5^ solar energy is a key and effectively limitless source of energy for powering such devices. However, the inconsistency of daily insolation means that to ensure autonomous operation, the solar energy harvesting aspect must be combined with an energy storage system to provide a continuous source of electricity during the operation cycle of the devices.^6−8^ Most solar cells have open circuit voltages of 0.6–1.0 V,^9^ which makes them unsuitable to be used directly with Li-ion batteries (LIBs) without dc-to-dc power converters, or stacking.^6,8^ Similarly, at the utility scale, the output of a string of solar modules requires expensive power electronics to couple the energy-harvesting and storage infrastructures. Much work has been done on tackling this problem such that ohmic losses and packing inefficiencies between the two devices can be reduced; however, ongoing manufacturing challenges, which ensure performance without increasing the balance of plant costs, remain.^8^ An ideal solution would be the development of photoelectrodes that can harvest solar energy and store it natively without the need for energy converters.^10−12^ Such a technology could conceivably be implemented at the device scale or in off-grid rural settings, where autonomous operation and low maintenance are particularly desirable.

Several different strategies have been proposed to combine energy harvesting and storage. These include three-electrode photobattery cells, in which the energy harvesting and storage units are compartmentalized,^13,14^ as well as more integrated two-electode systems that have been applied to Li-ion,^15−17^ Zn-ion,^18^ and Ag-ion batteries.^19^ Two-electrode solar batteries rely on materials that can simultaneously harvest and store energy. In this configuration, photocharging has been demonstrated with organohalide perovskites,^16^ MoS_2_,^20^ and organic materials,^22^ with many more proposed or in early stages of study. One of the most promising materials that has emerged for photobatteries is V_2_O_5_, which has demonstrated the ability both to harvest solar energy and to store it in the form of a Li-ion battery (LIB),^17^ a Zn-ion battery,^18,21^ and even a photoactive Zn-ion capacitor.^20^ However, even with Li_*x*_V_2_O_5_, the overall power conversion efficiencies of photobatteries have remained low, particularly in terms of the maximum charge state that can be achieved under illumination. To begin to overcome this, a better understanding of the photocharging mechanism is required.

The hypothesis as to how photobatteries work mechanistically has been founded on that of a heterojunction solar cell.^8,10,17−19^ Photoexcited charges are generated under illumination and then separated by the presence of hole/electron transport layers neighboring the photoactive electrode material. The electrons are transported to the anode to recombine with Li^+^ ions to form Li^(0)^, triggering the release of intercalated ions from the cathode;^18^ this process, however, is energetically challenging to justify. An alternative explanation is that the photo-generated electrons react to form a solid–electrolyte interphase.^15^ While an attractive idea, it is for instance unclear how this can explain decrease in impedance observed during cycling under light.^17^ Consequently, the question of whether photocharging effects are “real” or if the measured effects are the result of a series of side reactions and thermal effects remains unanswered.

Here, we use optical reflection microscopy to track the lithiation and delithiation in individual 2–15 μm polycrystalline Li_*x*_V_2_O_5_ particles and directly interrogate the photocharging mechanism. We find that exposure to light for which *E*_photon_ > *E*_BG_ (where *E*_BG_ is the band gap energy of the material) results in polarization of the Li concentration within the electrode particles, which is absent with sub-band gap illumination or upon heating of the cell. Our results show that light can drive changes in lithiation of Li_*x*_V_2_O_5_ and indicate hitherto unseen surface and subsurface effects in the particles that may allow charge neutrality to be maintained. More generally, our work demonstrates an optical methodology for robustly interrogating charge dynamics in photobattery electrode materials, which is essential for their study and optimization.

Figure 1a schematically shows the wide-field optical reflection microscope setup used in this study. It comprises a filtered (650–900 nm) white light excitation source, a 515 nm light-emitting diode (LED) for photobattery illumination at the Li_*x*_V_2_O_5_ band edge, and a CMOS detector. The cell is mounted in the microscope and connected to a potentiostat to control the electrochemistry of the cells while monitoring their optical properties in real time. Like many oxide materials, the visible (400–800 nm) reflectivity of Li_*x*_V_2_O_5_ changes in both spectral position and intensity drastically with the state of lithiation.^23,24^ Specifically, in Li_*x*_V_2_O_5_, the changes in reflectivity with the lithium fraction are suggested to arise from varying degrees of tilting of the VO_6_ octahedra and V 3d_*xy*_–O 2p_*x*_/2p_*y*_ orbital overlap in the different lithiation states.^25,26^ While such changes in reflectivity with lithiation state are known, the spatial pattern in which they occur on the micrometer scale particles, a first step in understanding the (de)lithiation mechanism, has yet to be established. Wide-field optical microscopy is therefore an ideal tool for investigating lithiation dynamics in this system (and many others^27−30^). The drawbacks of the technique are that it is restricted to probing two-dimensional dynamics and particles on the far side of the cell separator where not all of the material may be active and electrolyte depletion effects may be accentuated (see Supplementary note 1 for mitigation approaches). Furthermore, quantitatively linking spatial reflection intensities to the material (de)lithiation state remains challenging using optical microscopy alone, with caution needed when comparing interagglomerate behavior.

**Figure 1:**
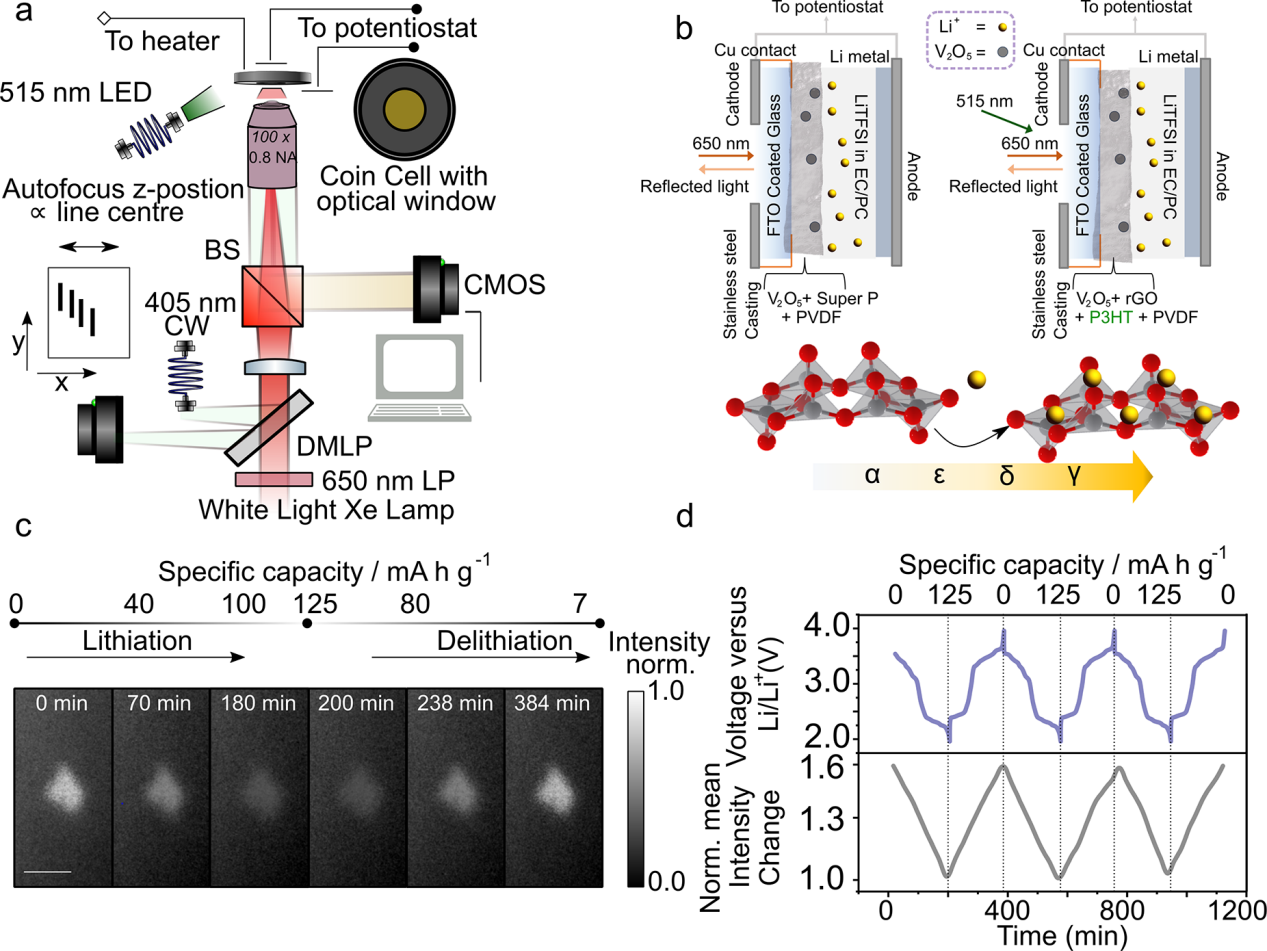
High-resolution optical imaging of an electrode particle during repeated charging and discharging of a Li_*x*_V_2_O_5_ lithium-ion battery. (a) Schematic representation of the optical setup used for *operando* electrode particle imaging. A coin cell with optical access allows for simultaneous cycling of the battery and imaging of the electrode under a variety of external conditions, e.g., light soaking or heating. A line-based autofocus ensures continuous imaging of a fixed plane (see Supplementary note 1). The wavelength of Illumination/reflection light is from 650 to 900 nm. A high numerical aperture (0.8 N.A.) long-working distance objective sets the lateral spatial resolution to ∼500 nm (full width at half-maximum) and the axial resolution to 1.8 μm. DMLP is a long-pass dichroic mirror, LP a long-pass filter and BS is a beam splitter. (b) Cell assembly under normal potentiostat-driven charge/discharge cycling (top left) and photobattery cell under light-enhanced charge/discharge cycling with added charge transport layers (top right). An additional 515 nm (>*E*_BG_ of Li_*x*_V_2_O_5_) LED light source is used to photoexcite electron hole pairs in Li_*x*_V_2_O_5_. Li_*x*_V_2_O_5_ undergoes phase transformations^31,32^ from α/ε to ε/δ to δ/γ phases with an increase in lithium concentration (bottom). (c) Optical reflection microscope images of an individual Li_*x*_V_2_O_5_ particle at selected charge states during lithiation and delithiation. The particle dims upon lithiation (discharge) and brightens upon delithiation (charge). The scale bar is 5 μm, and the intensity is normalized to the brightest pixel across the entire image stack. The signal-to-noise ratio across the stack is between 3 and 8 with a standard deviation of ∼0.03 on the normalized pixel intensity. (d) Discharge–charge curve (top, blue) for the battery as a function of time, along with the corresponding mean normalized change in the image reflection intensity of the particle shown in panel c (bottom, gray). The intensity is obtained by averaging across all pixels that correspond to the particle (see Supplementary note 4) and, as per previous studies,^33,34^ is normalized to the intensity at the point of lowest potential in the cycle.

We use electrodes made with the Li_*x*_V_2_O_5_ active material (∼90% loading) in a matrix of Super P conductive carbon and a poly(vinylidene fluoride) (PVDF) binder in the custom coin cell geometry shown in Figure 1b. A transparent FTO-coated glass window allows for optical access to the electrode and also acts as a current collector. To probe the light-induced effects within the Li_*x*_V_2_O_5_ particles, the electrodes are modified by mixing the Li_*x*_V_2_O_5_ active material with P3HT and rGO, which act as electron transport layers^17^ (see Supplementary note 1 for fabrication).

Figure 1c shows typical bright-field reflection images, normalized to the brightest pixel across the entire cycle of a Li_*x*_V_2_O_5_ particle, during a discharge (lithiation)/charge (delithiation) cycle. We note that although there is a range of particle sizes on the electrode (which may exhibit a range of charging dynamics depending on the size), our studies are limited to 2–15 μm particles that are relatively isolated from one another (∼5 μm separation) for ease of identification and analysis (see Supplementary note 2 for additional data sets). Upon lithiation, a decrease in reflection intensity (at 650–900 nm) is observed, whereas upon delithiation, the intensity of the light reflected from the particles increases. This in agreement with spectral reflectivity measurements (see Supplementary note 3) that show an increase in the percentage reflectance during the charging of bulk Li_*x*_V_2_O_5_ electrodes (removal of lithium) at wavelengths of 650–900 nm.^17,23^ Repeated imaging over multiple cycles, as shown in Figure 1d, where the normalized reflectivity change across all pixels contributing to the particle is plotted, demonstrates that the intensity of the light reflected from the particle correlates well with the battery state of the charge/electrode lithiation state. Although the specific capacity reports on the bulk electrode behavior whereas imaging is on individual particles, the use of relatively low current densities (∼1.6–2.3 mA cm^–2^) means we do not expect large discrepancies in the state of charge between particles. In general, we find that of the 63 particles we image, >85% are electrochemically active and show similar behavior as detailed in Figure 1c . Furthermore, we find our optical coin cells to be electrochemically robust with similar spatial (de)lithiation dynamics observed even after 100 cycles (see Supplementary note 3).

To analyze (de)lithiation under galvanostatic conditions, we determine the differential intensity contrast across a cycling particle over time. This quantity, which we denote as *ΔI*, is taken as , where *I*(*t*_*i*_) and *I*(*t*_o_) are normalized intensity images at a given point, *t*_*i*_, and at the start, *t*_o_, of the cycle, respectively, i.e., when a cell is at its maximal state of charge (see Supplementary note 4). The differential imaging technique allows us to remove background contributions and inhomogeneities in the illumination by isolating and subtracting changes between the images.^33,35^ Before calculation of *ΔI*, the normalized intensity images are thresholded (see Supplementary note 4) to remove further noise and nonparticle contributions. A negative (positive) differential contrast indicates a decrease (increase) in reflectivity compared to that of the particle in its maximal charge state.

Figure 2a shows the differential intensity image (*ΔI*) of the Li_*x*_V_2_O_5_ particle considered in Figure 1. During lithiation (discharge), negative differential intensity grows from the edges of the particle toward the center, indicating an inward propagation of a lithium-rich phase.^35^ Conversely, during delithiation (charge), a front of positive intensity, corresponding to areas of lithium loss, is observed to move in the same direction. We note that at the start of the cycle the differential contrast at the particle center is very slightly positive (20 min image); however, this is likely due to illumination variations or small lateral particle movements (see Supplementary note 4 for correction methods). We collect light from an axial extent of ∼1.5 μm inside the particles; hence, our signals are a projection of the surface and bulk dynamics of particles (see Supplementary note 5 for a discussion of penetration depth). While particles do have a degree of positive curvature, given that the entire particle can be placed in focus in a single plane and the particle periphery can sometimes be brighter than the center, i.e., in opposition to what would be expected on the basis of signal path lengths alone, we can discount the significance of such effects. In Supplementary note 4, we repeat measurements in several different focal planes to ensure our observations do not arise from particle curvature or refractive index mismatch effects, affording qualitatively similar behavior as in Figure 2a.

**Figure 2:**
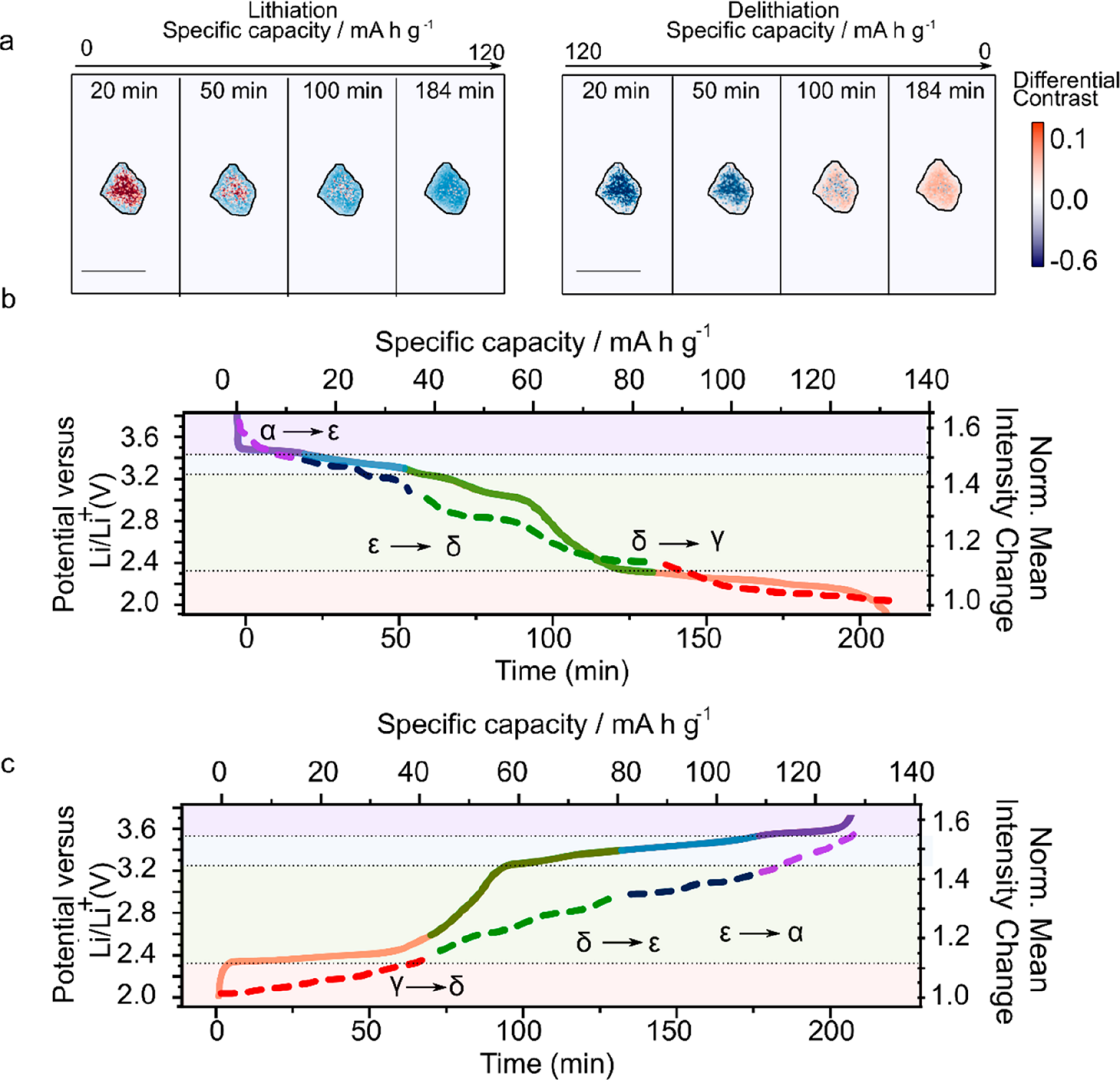
Optical tracking of phase transitions induced by lithiation and delithiation in Li_*x*_V_2_O_5_. (a) Differential contrast images of the same particle in as shown in Figure 1c as a function of the state of charge (left, discharge; right, charge). Raw intensity images are first normalized, background corrected, and thresholded (all pixels with a normalized intensity of <0.22 set to zero) before calculation of the differential contrast image (see Supplementary note 4). The intensity front grows from the edge of the particle to the center. The scale bar is 5 μm. (b and c) Specific capacity (solid line) during galvanostatic discharge (top) and charge (bottom). The corresponding normalized reflection intensity change (with respect to the lowest point of charge) averaging across all pixels of the particle is shown as a dashed line. Li_*x*_V_2_O_5_ is known to exist in different phases,^31^ α, ε, δ, and γ, in the voltage ranges specified by the purple, blue, green, and red shading, respectively.

During both charge and discharge processes, the main changes in lithium concentration are occurring around a central region of the particle with a distinct shell developing after 50 min of the cycle. After 50 min, the differential contrast dissipates to form a more homogeneous region across the entire extent of the particle, indicating that from this point, changes in lithium concentration occurralmost homogeneously throughout the whole visible extent of the particle. The behavior displayed in Figure 2a is consistent across particles in the electrode and over multiple cycles (see Supplementary note 4). Finally, we note that during lithiation/delithiation Li_*x*_V_2_O_5_ is known to undergo several phase transitions.^31,32,36,37^ In our data, we demarcate these transitions in the potential and time plots of Figure 2b with dotted horizontal lines. While the change reflectivity is monotonic with potential, as revealed also by spectral reflectivity measurements (see Supplementary note 3), it is not necessarily linear; e.g., there is a small change in reflectivity with potential in the δ phase that is likely due to the electronic properties of the underlying phase itself. Furthermore, while the differential intensity over the entire particle and differential voltage with respect to time appear to be correlated (see Supplementary note 6), suggesting our experiment is indeed sensitive to phase boundaries, additional work is required to resolve these fully.

Having established the ability of optical reflection microscopy to track the intercalation/deintercalation of Li-containing phases in V_2_O_5_, we apply the technique to study (de)lithiation under photocharging conditions. Experiments are repeated in the same configuration as shown in Figure 1a, but the electrode is modified by adding 1% P3HT and 1% rGO to the composition, to act as electron transport layers and provide a pathway for charges to be removed from the electrode, as required to enable the photocharging effect.^17^ The energy level diagram of the Li_*x*_V_2_O_5_ photocathode under photocharging conditions is shown in Figure 3. A summary of the contemporary model for photocharging can be broken down as follows. Photons of an energy greater than the band gap of the active material are incident on the electrode and result in the excitation of an electron–hole pair. The electrons are extracted due to the inclusion of transport layers, with the energy levels designed such that an energetically favorable pathway for the electrons to exit the electrode is created. The remaining holes are suggested to result in the oxidation and removal of the intercalated Li species.^8,10,20^ To date, some photocharging processes have been carried out under an open circuit condition, which should forbid the motion of the electron from the cathode to the anode. However, in these studies,^16,18^ a clear increase in cell potential and even additional charge capacity are observed. Here, we endeavor to reconcile these two effects by imaging the Li-ion-containing phase dynamics within individual Li_*x*_V_2_O_5_ particles also under illuminated galvanostatic cycling.

**Figure 3:**
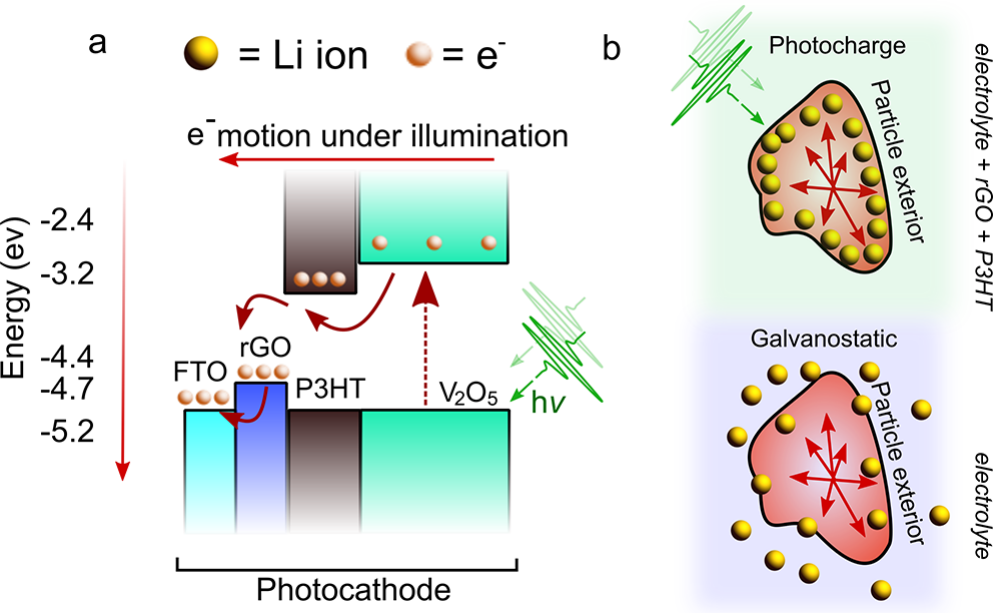
Electron and lithium-ion dynamics under photocharging and galvanostatic charging. (a) Schematic energy level diagram of the Li_*x*_V_2_O_5_ photocathode under the photocharging condition. Extraction of electrons via the transport layers (P3HT and rGO) results in the buildup of holes and subsequently Li ions on the exterior of the Li_*x*_V_2_O_5_ particle. (b) Schematic illustration of particle level dynamics. Under illumination, the Li concentration increases at the electrode subsurface (top). Under normal (dark) operating conditions, the charge current from the potentiostat facilitates total charge compensation at the anode and cathode sides (bottom). Lithium is deintercalated from the Li_*x*_V_2_O_5_ electrode particles.

Under photocharging conditions, a 515 nm LED (power of ∼150 mW; ∼10 mW cm^–2^ intensity at the sample) is used to illuminate the entire electrode. Any reflected light from the charging LED is rejected from the camera with appropriate filters, and the imaging is again performed from 650 to 900 nm. The source used for imaging is sub-band gap and hence does not induce any photocharging effects itself.^38^ To begin, the cells are galvanostatically discharged to 2.2 V. This is followed by measurement of the open circuit voltage under two conditions: with LED illumination and without (dark). At open circuit conditions it is assumed that it is not possible for charges to travel completely from the photocathode to the Li anode. Therefore, in this configuration, we specifically image what we understand to be the initiation of the photocharging process, namely the removal of electrons and redox-induced delithiation from the Li_*x*_V_2_O_5_ particles.

In Figure 4a, differential contrast images at given time intervals (with *t*_o_ taken when the cell is at 2.2 V) are plotted for single Li_*x*_V_2_O_5_ particles while measuring the open circuit potential (OCP) without LED illumination. In the absence of LED illumination, little change is observed in the differential contrast over time, with just a small degree of brightening (∼5%) of the image concomitant with an increase in the OCP from 2.2 to 2.4 V over 500 min. This small change could be a result of small focus fluctuations and thermodynamic relaxations within the cell. From 500 to 2000 min, both the reflected intensity from the particle and the OCP remain constant. The particle edges in the differential image are slightly negative likely to refractive index mismatches between the particle and matrix.^39^

**Figure 4:**
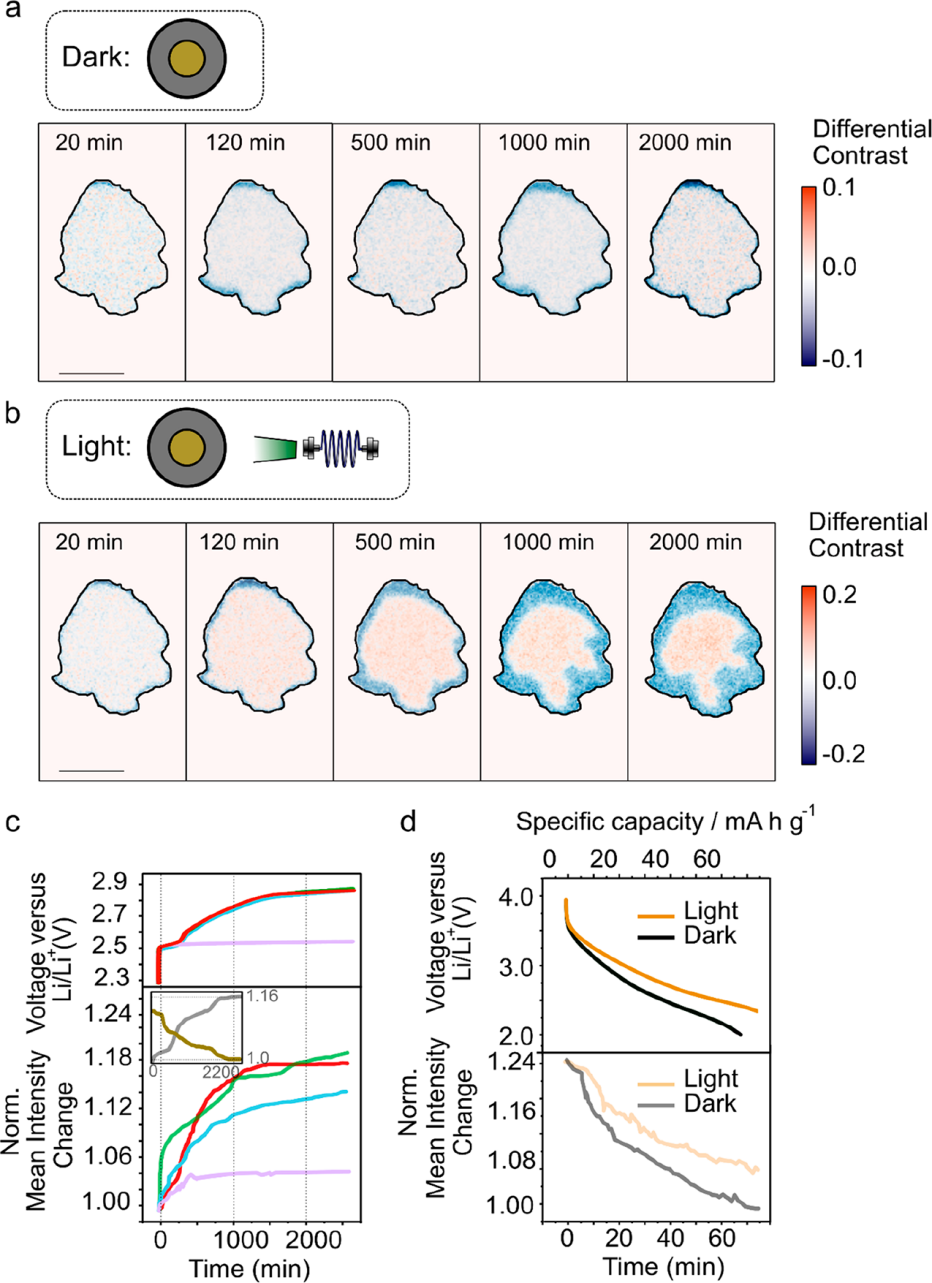
Optical imaging of Li_*x*_V_2_O_5_ particles during normal and photocharging cycles. (a) Differential contrast image of a Li_*x*_V_2_O_5_ particle during open circuit potential measurement with no additional light source. In the normalized reflectivity images, pixels below an intensity of 0.29 are set to zero before calculation of the differential contrast. (b) Differential contrast image of the Li_*x*_V_2_O_5_ particle during an open circuit potential measurement with illumination from a 515 nm LED. The scale bar in both panels a and b is 5 μm. The same thresholding as in a is used for these images. (c) Open circuit potential (top) and normalized reflection intensity change (bottom) at the center (1 μm diameter circle from the particle center-of-mass) of the particle shown as a function of time. Red, green, and blue curves represent multiple photocharging cycles. The purple curve represents the dark control condition. The inset shows the change in intensity of the core (gray) and outer (gold) regions of the particle. The core is defined by a 1 μm diameter circle at the particle center of mass, while the periphery is defined by a 1 μm wide strip defining the particle periphery (see Supplementary note 4). (d) Change in the potential and normalized reflected intensity (from the particle shown in panel a) averaged across all pixels that make up the particle as a function of the time and charge state, during galvanostatic discharge with the light on (gold) and off (gray). When the light is on, the discharge is slower, which is also reflected in the change in intensity.

When the battery is exposed to the green LED light, however (photocharging condition), a significant decrease in the differential contrast (darkening) is observed at the edges of the particles that is concurrent with an increase in contrast (brightening) at the center, as shown in Figure 4b. Under these conditions, there is an ∼18% increase in the normalized (with respect to image at 2.2 V) intensity of the particle center (averaging pixels in a 1 μm diameter circle at the particle center of mass (see Supplementary note 4)) throughout the photocharge. The measured OCP also increases from 2.2 to 2.9 V during 2000 min of light exposure, consistent with previous studies.^17,21^ On the basis of our previous observations, an increase in the differential contrast suggests that there is a local decrease in the Li-ion concentration, i.e., lithium-poor phase at the particle center, and the dimming at the periphery indicates an increase in Li-ion concentration at the edges of the particle, i.e., lithium-rich phase. This would imply that light exposure drives lithium from the center to the edges of the Li_*x*_V_2_O_5_ electrode particles. The behavior can be reproduced over multiple cycles, suggesting that irreversible side reactions play a limited role in the behavior. Furthermore, changing the photocharging wavelength to 480 nm (and decreasing the photocharging light source intensity to 6 mW cm^–2^; setup constraints limit variation beyond this) results in qualitatively similar behavior, tentatively suggesting that the precise energy of photons does not play a significant role but requires further investigation (see Supplementary note 7).

The results presented above cannot be explained in the case of an isolated particle of Li_*x*_V_2_O_5_, suggesting that the additional elements of a photoelectrode composition, namely, the electron transfer layer, play a key role in the effect. Indeed, repeating experiments without an electron transfer layer (Supplementary note 7) does not result in behavior like that detailed in Figure 4. This is in accordance with previous studies.^18,38^ A mechanism that has the potential to invoke this effect is the transfer of photoexcited electrons from Li_*x*_V_2_O_5_ to the P3HT electron transport layer and the subsequent motion along the induced concentration gradient of lithium ions to the exterior of the particle. Under illumination, we observe the buildup of a Li-rich phase on the exterior of the particles, which we would expect to lead to a lower cell voltage. However, we detected an increase in OCV (in accordance with all previous studies), which leads us to believe that Li ions are leaving the active material because delithiation would correspond to an increase in the state of charge of the battery. Because under the OCV the electrons cannot travel from the cathode to the anode, a logical explanation would be that the generated electrons and Li ions are stored at the electrode surface (see the schematic in Figure 3).

The net intensity changes of the core region (gray) and edge region (gold) are plotted in the inset of Figure 4c (see Supplementary note 4 for the definitions of regions). As already determined, the intensity of the core region increases during the photocharging process, indicating a reduction in the relative lithium concentration. Conversely, the intensity of the outer ring decreases, indicating a local increase in the lithium concentration. The gray line is seen to increase to a slightly larger magnitude than the gold line decreases, indicating that the sum over both regions (core and outer ring) results in a net loss of lithium; i.e., more lithium is lost by the core than is gained by the periphery. This must imply the loss of lithium to the volume surrounding the particle, in agreement with our proposed explanation described above. If lithium is indeed lost to the surrounding electrolyte, this would necessitate a transfer of charge from the electrolyte to Li_*x*_V_2_O_5_ for charge compensation, which in turn would originate from the lithium metal anode. The overall effect is that of a genuine recharge mechanism.

“Photo-enhanced” is the term that has been used to describe the condition in which a cell is controlled via a potentiostat, but with light-induced effects bolstering some performance metrics of the overall device such as the discharge capacity and capacity retention at higher current rates.^15,18,40^ In this work, we observe that during a galvanostatic discharge when the electrode is illuminated (the requisite conditions for photoenhanced operation), the reflected intensity and voltage both decrease more slowly than when the same electrode is discharged in the “dark” as shown in Figure 4d. Under these conditions, the external circuit is necessarily closed. Because the circuit is complete, charges may pass from one electrode to the other. Under illumination, the resulting discharge curve is less steep despite the applied current being the same as that under the dark conditions. This could be explained by the now permitted photocharging mechanism occurring constantly as a background process. The result is an increase in the observed gravimetric capacity of the cell; however, here we show how this does not result in an increased number of charges stored in the electrode. Rather, it could imply that charges are repeatedly being stored and removed under this condition. This mode of operation holds much promise and shows how photobattery devices could be used as a true combination of a solar cell and a battery under the condition that the drawn current (discharge current) is low enough, allowing the external load to be powered without depleting the corresponding amount of charge from the battery.

To investigate the proposed polarization model of the photocharging effect, we repeat the photocharging experiments, and after ∼900 min of photocharging under open circuit voltage conditions, we close the external circuit. As shown in Figure 5, the initial photocharge results in an overall brightening of the particle core and dimming at the edges. However, when the circuit is closed, the front of negative differential intensity recedes rapidly (relative to the charge process) and the overall intensity of the particle dims as highlighted in Figure 5b. In total, the normalized particle reflectivity (taking pixels in a 1 μm diameter at the particle center of mass) increases by ∼10% compared to its starting value during the photocharge, followed by a dimming by almost the same amount when the circuit is closed. Repeat experiments in which we apply −1 nA of current (the lowest setting available on the potentiostat used) produce a behavior similar to that observed in panels a and b of Figure 5, following the photocharge. However, in this case, the reflected intensity (and potential) does not drop immediately, and it takes ∼400 min for the reflection intensity across the particle to become uniform (see Supplementary note 8). Nonetheless, our results suggest that it is the act of closing the external circuit that results in the homogenization of the previously built-up polarized state across the particle.

**Figure 5:**
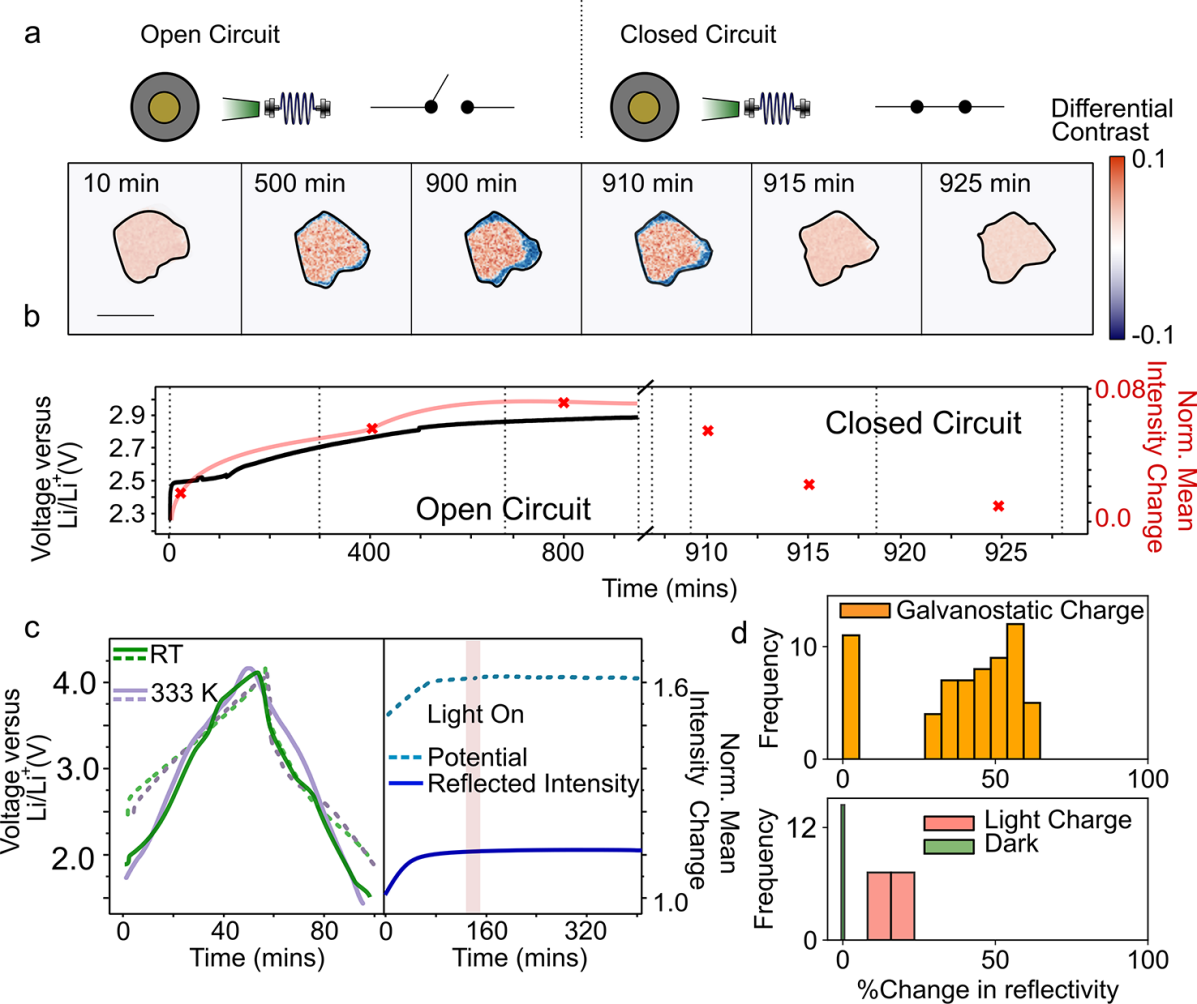
Examination of the photocharging polarization mechanism in Li_*x*_V_2_O_5_ electrode particles. (a) Differential contrast image (with respect to the image at 2.2 V) of a Li_*x*_V_2_O_5_ particle during an open circuit potential measurement with illumination from a 515 nm LED, followed by closing the external circuit at ∼900 min. Raw intensity images are first normalized, background corrected, and thresholded before the differential contrast image is calculated (see Supplementary note 4). The particle shape appears to (reversibly) change to a small degree throughout the charge and relaxation; however, within our limited spatial resolution, these changes are too small to separate from refractive index or lateral focus variations. The scale bar is 5 μm. (b) Potential (black) of cell and total normalized reflected intensity (red) from a 1 μm diameter circle at the center of a Li_*x*_V_2_O_5_ particle in panel a as a function of cycle time. Red crosses mark times at which images are shown. (c) Galvanostatic charging of the particle shown in panel a at room temperature (RT, green) and 333 K (purple) (left panel). Dashed lines indicate the potential, and solid lines indicate the change in reflected intensity. Charging (with no 515 nm LED illumination) followed by a potential hold at 4.0 V (with 515 nm LED illumination) (right panel). The dashed line indicates potential (light blue), and the solid line (dark blue) the normalized mean change in reflected intensity across all pixels contributing to the particle. Red shading indicates the start of the potential hold and the point at which the 515 nm LED is turned on. (d) Histogram of the percentage increase in the reflected intensity of particles under galvanostatic charging, photocharging, and dark (under open circuit potential) conditions (see Supplementary note 4 for the exact regions of particles defined for extraction of data). Under the galvanostatic conditions, >85% of the particles are found to be active and show a core–shell (de)lithiation pattern. The particles with zero reflectivity change are assumed to be in active “dead spots” within the electrode. The slightly poorer performance of our cell compared to that of typical coin cells likely arises from the unusual geometry required by introducing a window for optical access and the necessity for the unconventional FTO-coated glass as the current collector.

To verify that the observations are not driven by heating effects, we perform galvanostatic charging and discharging from 1.8 to 4.0 V at 333 K (60 °C) (Figure 5c; see also Supplementary note 7). In this case, the charge and discharge curves closely resemble one another regardless of the additional heat. There is a small change in the position of various potential plateaus and gradients, as expected from the increased temperature, but no effects resembling those from light are observed. Similarly, concomitant increases and decreases in the reflected intensity from the particle are observed when charging and discharging both at room temperature (RT) and at 333 K. This suggests that thermal effects can be neglected in the lithium-ion photocharging mechanism in this cell. Additionally, although vanadium oxide materials are known to undergo a transient phase transition^41^ (typically from a metal to an insulator) under strong pulsed illumination or deep ultraviolet illumination^42^ (for specially prepared films), the illumination powers and wavelengths we use here cannot facilitate this. Indeed, under prolonged light exposure at 515 nm, we observe no change in the reflectivity of native V_2_O_5_ films (see Supplementary note 3).

Finally, to verify that the effect is associated with lithium motion and is not an electrochemical or optical artifact, the cells are galvanostatically charged to 4.0 V forcing the completion of all delithiation processes, followed by exposure of the cell to the photocharging conditions (Figure 5c). No changes to the cell reflection intensity are observed in this instance, as expected, because no change in the lithiated state may reasonably be induced beyond 4.0 V. The results are repeated for multiple particles within multiple cells, and the statistical reproducibility is summarized in the histogram in Figure 5d. The data contained in the histograms do not compare the absolute changes in reflectivity between particles, as these may be also influenced by external factors such as the required imaging plane and exact particle size and/or orientation^39^ (see Supplementary note 4 for further discussion).

In summary, we use optical microscopy techniques to investigate the (de)lithiation processes in Li_*x*_V_2_O_5_ particles under a variety of electrochemical, photocharging, and thermal conditions. We interrogate the photocharging phenomena reported in previous studies and observe that the process consists of a buildup of a Li-rich phase on the periphery of the active material and a partial delithiation. These effects may result in a combination of faradaic and nonfaradaic charge storage mechanisms on the particle exterior. Finally, we demonstrate the dynamics of such electrodes under photoenhanced operation, whereby light may be used to replenish a cell as it simultaneously discharges, allowing for a greater amount of power to be potentially drawn by an external device. Our results provide a more accurate description of the microscopic effects of illumination on light-sensitive battery electrode materials and a methodology for robustly probing microscale charge dynamics in photobatteries.

Future studies should aim to investigate how the results presented here translate to other photobattery materials such as halide perovskites^22^ or layered nanomaterials,^20^ where both intercalation and conversion reactions may take place. While we have focused on one part of the photocharging mechanism, electron transport within the charge transport layers and electrolyte needs to be investigated further. In terms of using optical microscopy in batteries, obtaining a quantitative readout remains an important goal. This may be achieved by converting reflectivity values into refractive indices , utilizing chemically specific optical probes such as Raman^43^ or hyperspectral imaging (where reflection bands could be aligned with specific electronic transitions^44^), correlating optical reflectivity measurements with X-ray imaging,^45^ or combining optical microscopy with more complex electrochemical tools such as the galvanostatic intermittent titration technique to obtain robust insights into material diffusion coefficients.

## Supporting Information Available

Large-area optical images of Li_*x*_V_2_O_5_ electrodes, spectral reflectivity of electrodes, additional (simultaneous) photocharging/galvanostatic (dis)charging and optical imaging measurements, estimation of the light penetration depth in electrodes, correlation between the differential voltage and differential reflectivity, galvanostatic (dis)charging and imaging at increased temperatures, experiments of photocharging at different wavelengths, and optical imaging following photocharging and closing of the circuit (PDF)
