# Supplementary material for: Interrogating the Light-Induced Charging Mechanism in Li-Ion Batteries Using Operando Optical Microscopy
Source: Nano Lett. 2023 Aug 8;23(16):7288–96. doi: 10.1021/acs.nanolett.3c01148 (PMC10450808; doi:10.1021/acs.nanolett.3c01148)
Supplement: Supplementary file 1 — nl3c01148_si_001.pdf [file nl3c01148_si_001.pdf]

Interrogating the Light-induced Charging Mechanism in Li-ion batteries using *Operando* Optical Microscopy

[1,2]Raj Pandya*[ɫ], [2,3]Angus Mathieson[ɫ], [3,4]Buddha Deka Boruah, [1]Hilton B. de Aguiar and [3]Michael de Volder*

[1]Laboratoire Kastler Brossel, ENS-Université PSL, CNRS, Sorbonne Université, Collège de France, 24 rue Lhomond, 75005 Paris, France

[2]Department of Physics, Cavendish Laboratory, University of Cambridge, JJ Thomson Avenue, Cambridge CB3 0HE, United Kingdom

[3]Department of Engineering, University of Cambridge, Cambridge CB3 0FS, U.K.

[4]Institute for Materials Discovery, University College London, London WC1E 7JE, UK

*Correspondence to: rp558@cam.ac.uk, mfld2@cam.ac.uk

ɫThese authors contributed equally to the manuscript.

Supplementary Information

Supplementary note 1: Methods 2

Supplementary note 2: Optical images of $Li_xV_2O_5$ electrodes 4

Supplementary note 3: Spectral reflectivity and repeated cycling of $Li_xV_2O_5$ 5

Supplementary note 4: Further analysis and image processing methods 7

Supplementary note 5: Penetration depth of light 12

Supplementary note 6: Correlation between differential voltage and differential reflectivity 13

Supplementary note 7: Photocharging at 480 and 515 nm, attempted photocharging without electron transfer layers and galvanostatic charging at 60°C 14

Supplementary note 8: Optical imaging following photocharging and closing of the circuit with -1 nA of current 16

References 17

Supplementary note 1: Methods

Reflection microscopy

The microscope was a standard layout of an epi-detected optical microscope. Illumination was performed with a Xe white lamp and illumination/collection wavelengths were set to 650 – 900 nm using the appropriate filters. An additional 515 nm LED (Thorlabs) was coupled into the microscope for photocharging/enhancement experiments. A long working distance, 0.85 NA 100 × (Olympus) objective, set the spatial resolution of our imaging setup to ~500 nm, with an overall camera-field-of-view of 60 μm (1 fps recording frame rate). Additionally, a line-based reflection autofocus (CW 405 nm) on a second camera was used to mitigate focus drifts or any change in focus introduced by electrochemical cycling. The total illumination power on the electrode was always below ~10 mW/cm^{-2}. Temperature dependent experiments were performed by attaching a ceramic heater (Thorlabs HT19R) to the back side of the con-cell casing and using a home-made temperature controller to set the temperature. Before imaging the temperature of the cell was allowed to equilibrate for 3 hr, with the temperature verified by using a laser thermometer (RS) shone onto the electrode.

To avoid focus drift and electrochemically induced defocusing a line-autofocus method is used based on reflection of a reference beam. This is a method partially adapted from Ortega Arroyo *et al.*[1]. A reference beam taken from a 980-nm diode laser (CPS980S, Thorlabs) is focused *via* a dichroic mirror (DMSP900, Thorlabs) onto the back-focal plane of the objective. The reflected beam from the bottom surface of the of electrode is picked off after the objective and focused to a line using a cylindrical lens (F = 200 mm, Thorlabs) into a line and detected by a CMOS camera (Grasshopper, FLIR). The refresh rate for the focus control was set to 20 Hz to allow for a fixed focus position with standard deviation of less than 50 nm over a full experiment.

V_2O_5 electrode fabrication

V_2O_5 powder was purchased from Sigma-Aldrich and used without further purification. V_2O_5 electrodes were prepared by dispersing 80 mg of V_2O_5 powder and 10 mg of carbon additive super P in 4 mL of NMP (received from Sigma-Aldrich). The mixture was mixed for approximately one hour using a magnetic stirrer bar and then sonicated for a further hour. Thereafter, 10 mg of PVDF (Solef 6020) binder was added into the solution followed by further mixing for another 12 h. The electrode therefore comprised V_2O_5:SuperP:PVDF in the ratio of 80:10:10. This electrode solution was drop cast onto a conductive FTO coated glass substrate (received from Sigma-Aldrich, surface resistivity ∼7 Ω sq^{-1}) and dried at 120 ^{o}C under vacuum.

V_2O_5 photoelectrode fabrication

V_2O_5 photobattery electrodes were prepared by dispersing 91 mg of V_2O_5 powder, 1 mg of P3HT and 1 mg of rGO in 4 mL of NMP (received from Sigma-Aldrich). The mixture was mixed for approximately one hour using a magnetic stirrer bar and sonicated. Thereafter, 5 mg of PVDF (Solef 6020) binder was added into the solution followed by further mixing for another 12 h. The electrode therefore comprised V_2O_5:P3HT:rGO:PVDF in the ratio of 93:1:1:5. This electrode solution was drop cast onto a conductive FTO coated glass substrate (received from Sigma-Aldrich, surface resistivity ∼7 Ω sq^{-1}) and dried at 120 ^{o}C under vacuum.

Cell assembly

A custom-built coin cell with an optical window was made by drilling a hole of ~8 mm diameter into the casing of one side of a (CR2032) coin cell. The electrode was fixed over this hole and sealed around the perimeter using two-part epoxy (Evo-Stik). This was heated at 70 °C overnight to ensure the complete curing of the epoxy. Thin strips of copper foil were used to contact the FTO surface to the coin cell casing. The battery assembly was completed by adding a glass fibre separator and 1 M lithium bis(trifluoromethanesulfonyl)imide (LITFSI) in ethylenecarbonate/propylenecarbonate (EC/PC) as electrolyte. To minimise electrolyte depletion/accumulation, the electrode was fully soaked before assembly and the electrode film is kept as thin as possible at approximately 1 μm. A lithium metal chip

was placed on a stainless steel spacer and placed under a stainless steel spring before being sealed together in a crimping machine (pressure ~1000 psi).

Supplementary note 2: Optical images of $Li_xV_2O_5$ electrodes

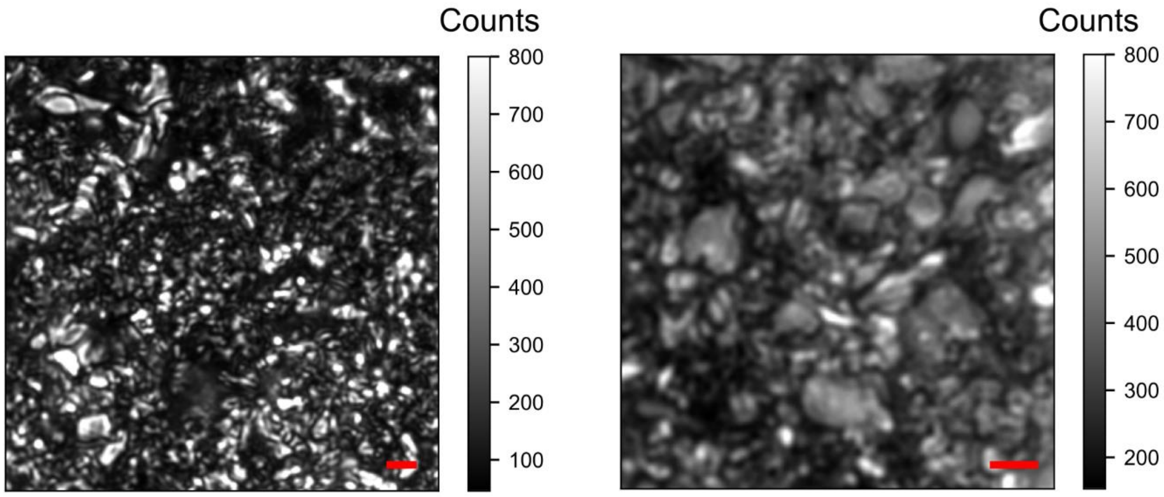

Supplementary Figure 1: Reflection image of dense regions of $Li_xV_2O_5$ electrode. In both images scale bar is 5 μm.

Supplementary note 3: Spectral reflectivity and repeated cycling of $Li_xV_2O_5$

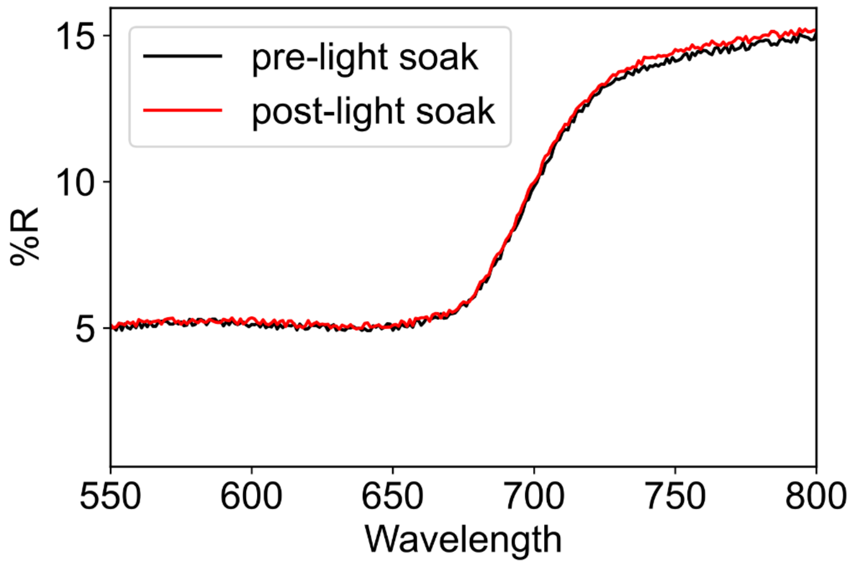

Supplementary Figure 2: Reflection spectra of pristine V_2O_5 before (black) and after light soaking for 3 hrs with 10 mW cm^{-2} 515 nm light. No significant change in the reflectivity is observed, suggesting that light-induced phase transitions cannot be induced in the native material under the conditions we use for study.

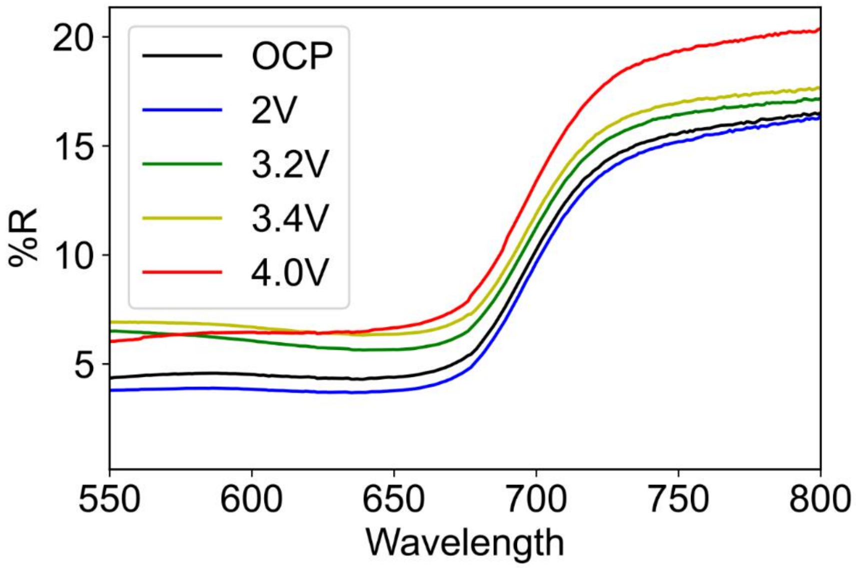

Supplementary Figure 3: Reflection spectra of pristine $Li_xV_2O_5$ as a function of voltage (vs Li/Li^+.). The reflectivity increases with potential as detailed in the main text and is monotonic but not linear in the 650 to 900 nm range we probe.

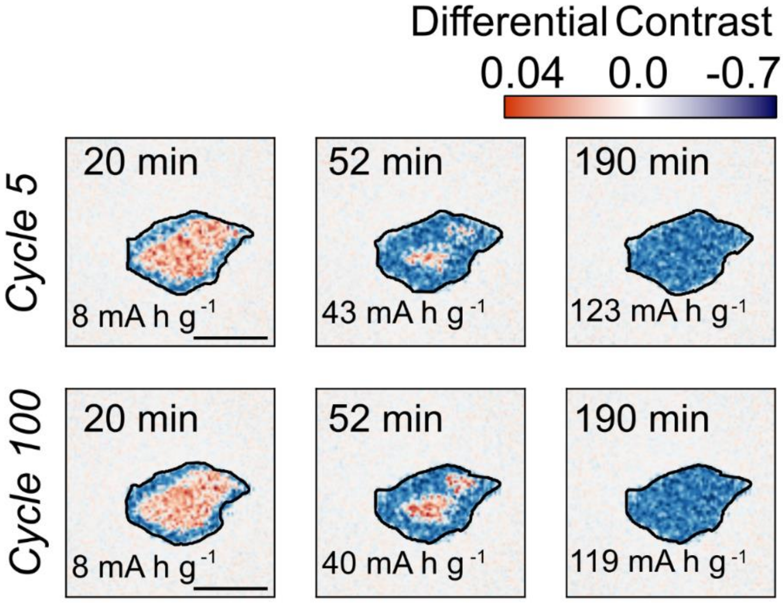

Supplementary Figure 4: Differential contrast images of a particle as a function of time for lithiation (discharge), without additional photocharging light, in the 5th cycle and 100th cycle. Raw intensity images are first normalised, background corrected and thresholded (all pixels with a normalised intensity below 0.2 set to zero) before calculation of the differential contrast image. The spatial reflectivity changes between the cycles is extremely similar both in magnitude of the changes and pattern which confirms the long-term stability of the electrochemical cells.

Supplementary note 4: Further analysis and image processing methods

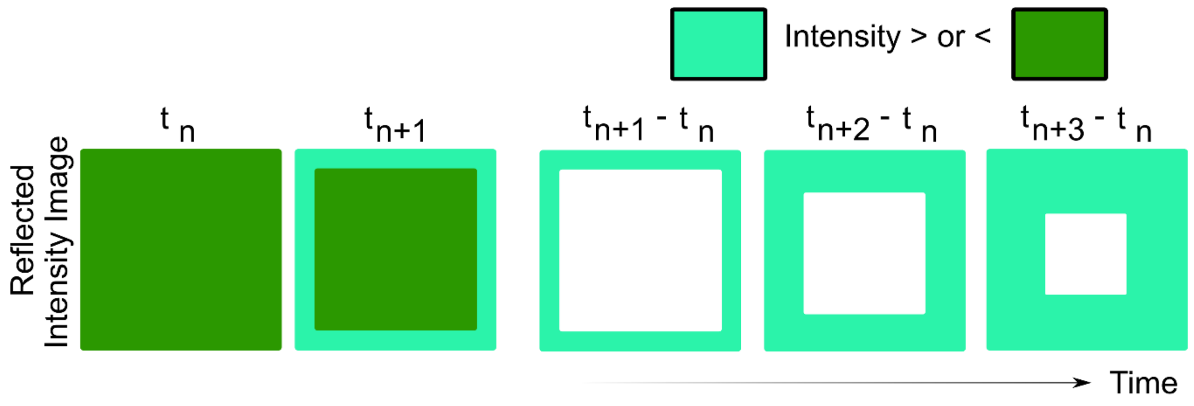

Supplementary Figure 5: Schematic illustration to describe how an increase or decrease in reflectivity of the particle from time t_n, to time, t_{n+i}, starting at the particle periphery, will give rise to a core-shell like intensity pattern when considering the differential reflectivity images.

In general: (i) when raw image intensities are presented we normalise data across the image stack, (ii) for differential images the contrast is taken with respect to the initial image at the start of the electrochemical cycle and (iii) when intensity trace plots are shown the data is obtained by averaging over the entire active particle during galvanostatic cycling and specific regions of the particle in the photocharging experiments (see below) with intensity normalized to the image intensity at the lowest potential in the charge-discharge cycle. This is an approach that has been followed by other groups presenting optical microscopy data of batteries[2–4].

The scientific justification for using the differential contrast method is that it allows us to remove background contributions and inhomogeneities in illumination to isolate changes between the images. We note that in some cases a pre-masking is applied to the differential images for clarity to exclude regions that are not part of the particle being visualised. In this case all pixels with a normalized intensity value below 0.1 and 0.31 (depending on the exact data set) are set to 0. This results in a differential background that is uniform and close to zero. Before processing of all imaging data a jitter correction is applied using the registration plug-in in ImageJ[5].

In order to define regions of the particle, both for evaluation of the background and determining the pixels which to include for deriving the intensity values, the Otsu thresholding method[6] was used to define pixels that correspond to the particle. This region was re-determined for each image to account for microscope drift and small changes in particle shape.

Estimating uncertainties on the semi-quantitative data extracted in our work is challenging. As shown by taking cross-cuts through the normalised reflection images the signal-to-noise ratio (as determined by the reflectivity of the particle compared to the background) varies from 3 to 8. The standard deviation on the normalised pixel intensities is ~0.03.

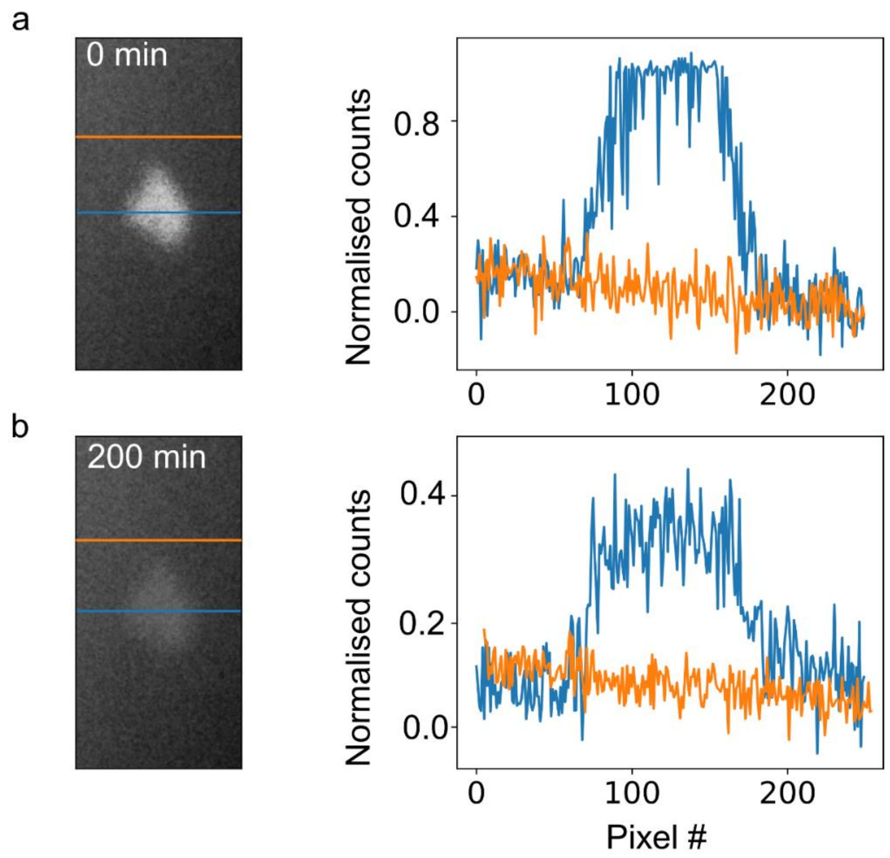

Supplementary Figure 6: a-b. Bright-field image of $Li_xV_2O_5$ particle at 0 min (a) and 200 min (b) during charge-discharge cycle and corresponding cross cuts along particle (blue) and background (orange) to give estimate of noise. The signal-to-noise ratio in images (taken from the magnitude of the reflectivity signal and background noise) is between 3 and 8 depending on the state of charge. The standard deviation on the normalised pixel intensity values is ~0.03.

We note that because we are using a long-working distance 0.8 N.A. objective our light-collection power is not as high as that reported in previous works using high 1.4 N.A. objectives[2,3]. However, as compared to these works we are less susceptible to refractive index mismatch based aberrations of focus related artefacts.

The choice of focal plane is crucial when performing optical imaging of battery materials. This is because of the often irregular shapes of battery particles and high-refractive index mismatch between the materials (n = 2 to 3) and the refractive indices microscope objectives are designed for (n = 1 to 1.4). Indeed, as we have previously shown inter-particle heterogeneities (which we do not comment on here) cannot be established from measurements at a single focal plane[7]. The choice of focal plane however becomes only very critical when using objectives with N.A.'s of ≤ 1. This is because although the refractive index mismatch is smaller for very high N.A. objectives, many spherical aberration effects scales as N.A.^{2} or N.A.^{3} [8]. It is for this reason in part we chose to perform experiments at a lower N.A. of 0.8 rather than with a high N.A. objective that could provide improved resolving power but potentially cause imaging artefacts.

To clarify the spatial reflectivity patterns we observe do arise from focus related effects, we repeated partial measurements at two different focal planes as shown below. No qualitative difference in the spatial patterns was observed. Furthermore, we performed checks of partial cycling without the autofocus to show that the autofocus feedback in this case does not contribute to the patterns we observe.

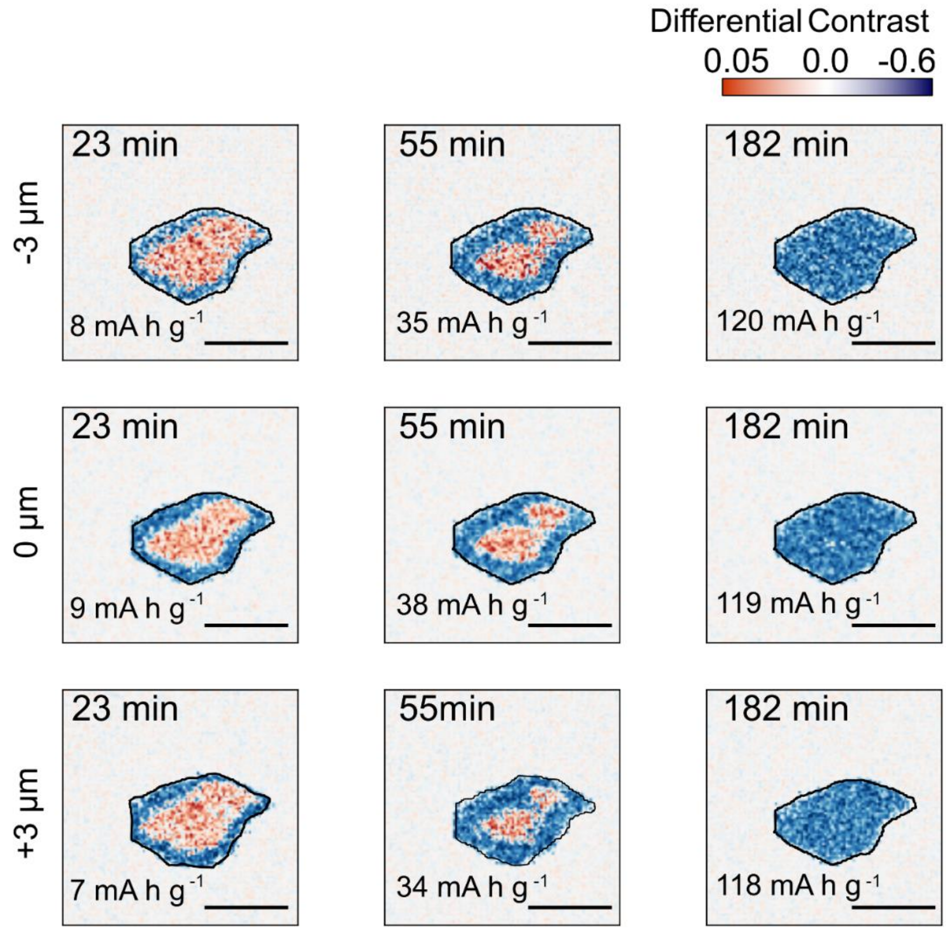

Supplementary Figure 7: Differential contrast images of a particle as a function of time for the 6th, 9th and 11th cycles during lithiation (discharge). The focus position of our objective is locked to relative positions of 3, 0 and -3 µm (which are separated by approximately 1.5 times the depth of focus, *i.e.* we are locking to different focal planes). Raw intensity images are first normalised, background corrected and thresholded (all pixels with a normalised intensity below 0.21 set to zero) before calculation of the differential contrast image. The spatial reflectivity changes between the cycles is extremely similar both in magnitude of the changes and pattern, with some slight blurring between focal positions being the main difference. Scale bar is 5 µm.

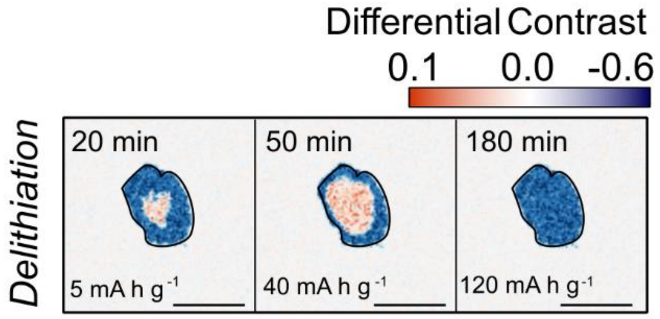

Supplementary Figure 8: Differential contrast images of a second particle as a function of state of charge. A similar behaviour to that observed in **Figure 2** of the main text is observed for other particles. Scale bar is 4 μm.

In the analysis of our photocharging effects in **Figure 4** and **5** of the main text we define specific regions of the particle to average the pixel intensity over and determine the reflectivity changes. For the periphery of the particles a custom image processing method[7] is used to define a strip of width 1 μm that traces the exterior of the particle (see below). This strip is then used for determining reflectivity changes in all images. For the centre of the particle, the particle centre-of-mass is first defined as $\gamma_{COM} = \frac{\sum_i \gamma_i \times m_i}{\sum_i m_i}$ where γ is the x or y coordinate. A circle of 1 μm diameter is then defined at the centre-of-mass and the pixel intensity over this circle (weighting partial pixels appropriately) is calculated. This same approach is used for all particles when analysing photocharging behaviour. The choice of strip diameter is chosen to allow for a good balance between signal-to-noise in the determined change in reflection intensity, whilst making sure pixels at the centre and periphery do not overlap. Empirically we find that the behaviour to be tolerant to a range of strip widths and circle sizes. Furthermore, as we do not make quantitative comparison between data sets the magnitude of the change in reflectivity is not significant.

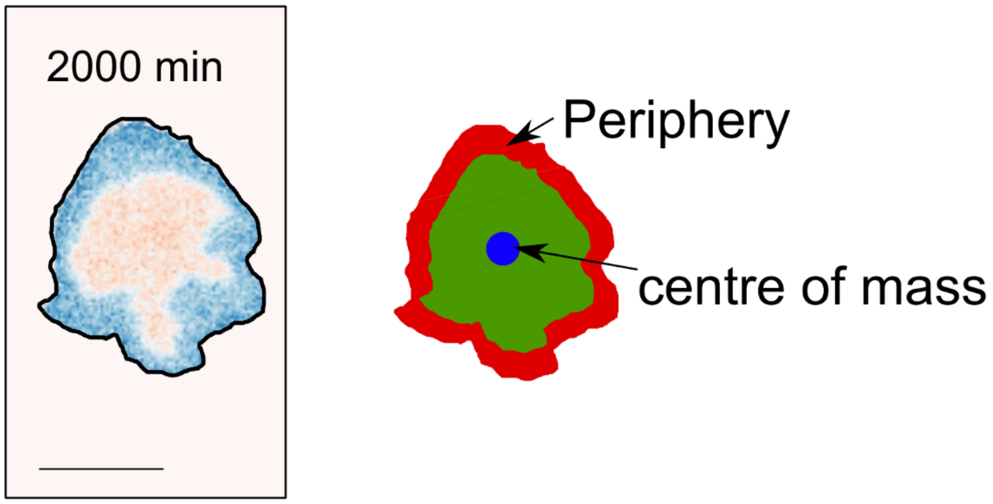

Supplementary Figure 9: Schematic highlighting how different regions of the particle are chosen for averaging pixel intensities over when defining the periphery and core in **Figure 4** and **5** of the main text.

Supplementary note 5: Penetration depth of light

To understand our optical images we must first consider what volume of the particles the reflected signal arises from, which itself is governed by the balance of the light attenuation through the sample and the lateral/axial resolution[8,9]. The lateral resolution $\Delta x = \Delta y$ of an optical microscope is typically estimated from the formula $\frac{0.61 \cdot \lambda}{NA}$, where NA is the numerical aperture of the optical system (0.8) and λ (650 nm) is the centre frequency for imaging in our system. For our system $\Delta x = \Delta y$ ~495 nm. For widefield detection the axial resolution is defined as $\frac{1.77 \cdot n \cdot \lambda}{NA^2}$ where n is the refractive index of the immersion medium (air in our case; n = 1). For our system the axial resolution is which for our system would be ~1.5 to 1.8 µm.

$Li_xV_2O_5$ is relatively transparent material (see photo in **Supplementary Figure 10a**). By moving the focus through a thick portion of the sample we can see that the signal only drops by $1/e$ of its initial value after about 12 µm, *i.e.* six times the axial resolution (**Supplementary Figure 10b**). Assuming particles of thickness between 5 to 10 µm (as previously measured by SEM[10–12]) we can see that our signal can have contributions from the particle volume, *i.e.* both the surface and part of the bulk. Hence what we observe is a 2D projection of the overall bulk and surface signal across agglomerates.

We note that distinct patterns of intercalation, such as core-shell, have been previously reported in agglomerate materials[13] which can be similar or different to those observed at single crystal particle level[14,15].

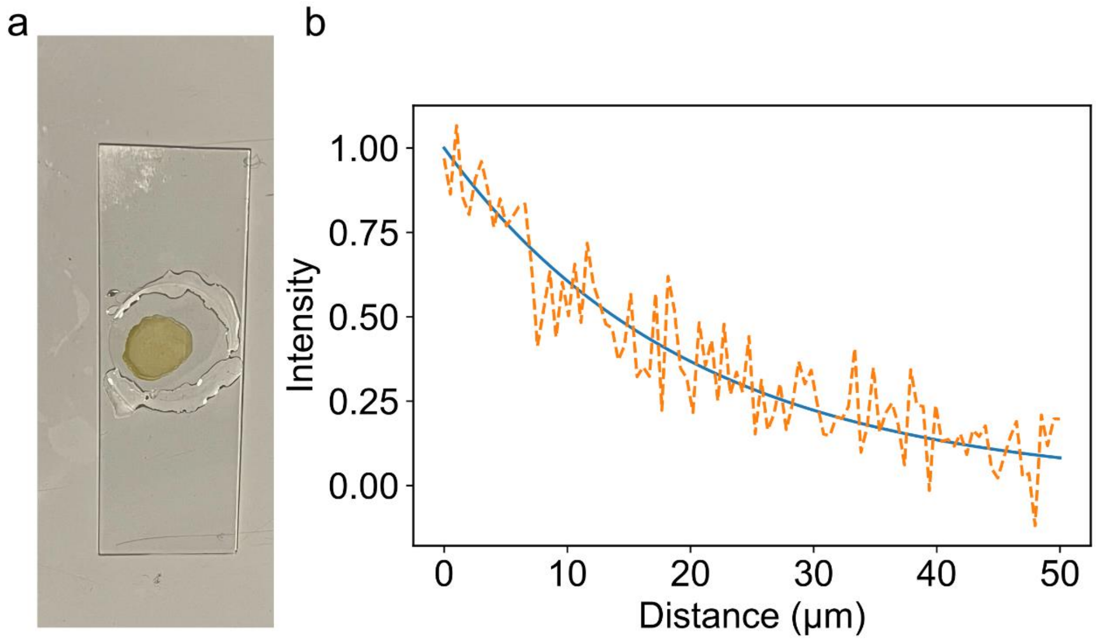

Supplementary Figure 10: a. Photo of ~100 µm thick bare V_2O_5 agglomerates film showing the material to be semi-transparent. **b.** Exponential decay of reflected white light from centre of an agglomerate. Orange line shows raw data and blue line fit. From the point where the intensity drops by $1/e$ of its initial value the attenuation of the material can be estimated which is ~15 µm, *i.e.* 5 to 6 times that of the axial resolution.

Supplementary note 6: Correlation between differential voltage and differential reflectivity

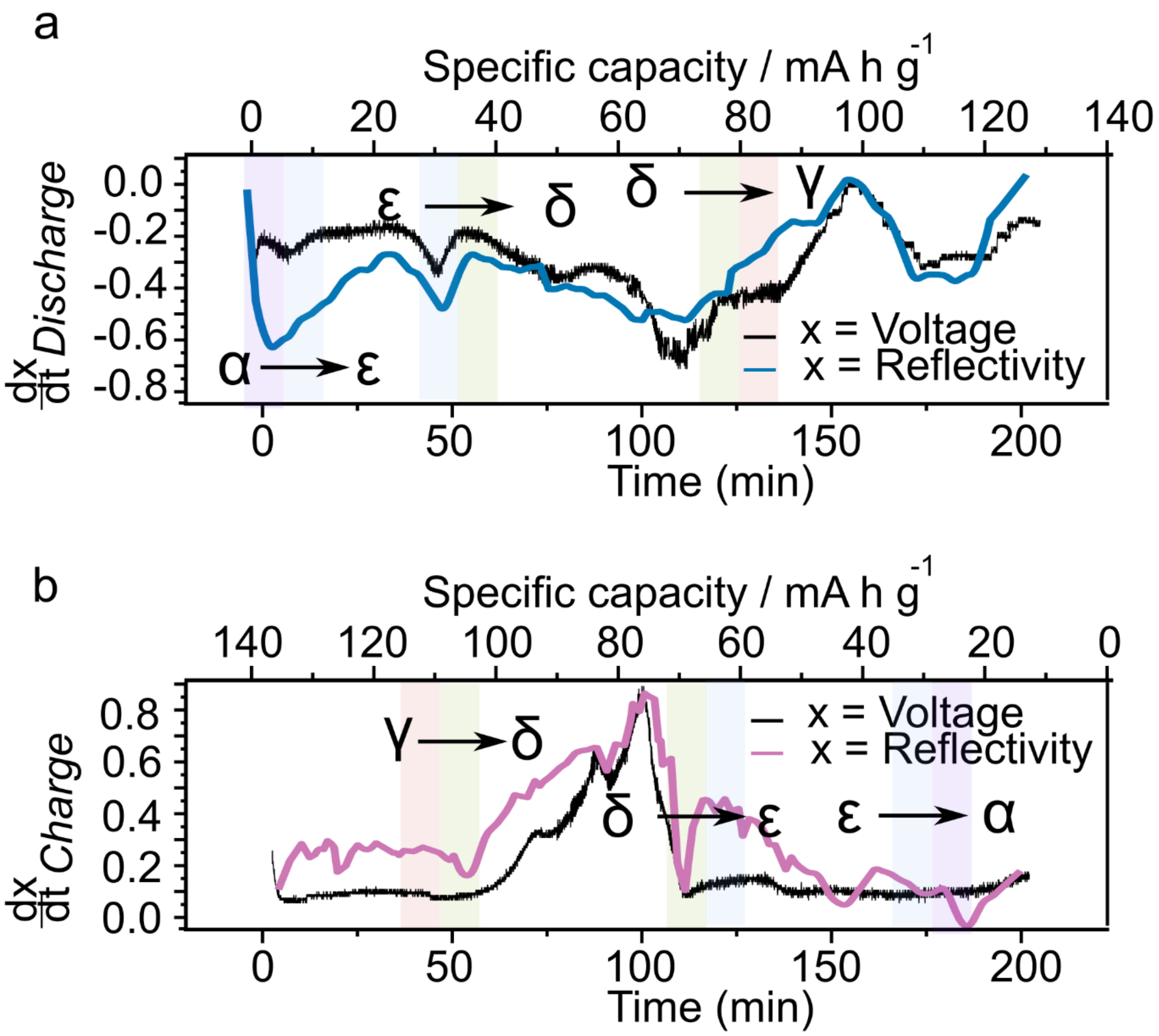

Supplementary Figure 11: Differential voltage and reflectivity from individual $Li_xV_2O_5$ particles as a function of charge state for both lithiation and delithiation. At the phase transition (highlighted in coloured shading) there are peaks that are correlated (Pearson R ~0.67) between $\frac{dV}{dt}$ and $\frac{dI}{dt}$ during both the charge and discharge.

Supplementary note 7: Photocharging at 480 and 515 nm, attempted photocharging without electron transfer layers and galvanostatic charging at 60°C

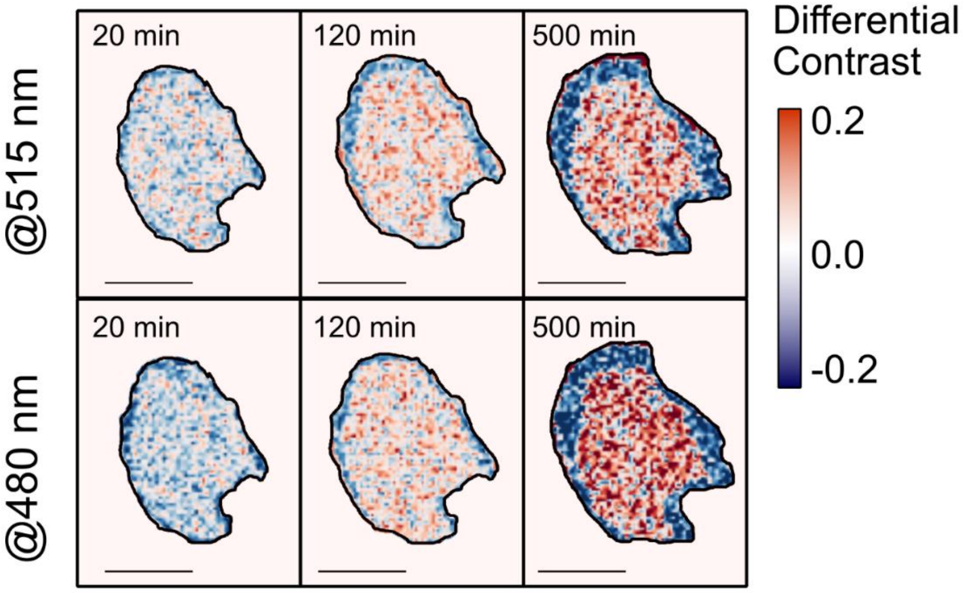

Supplementary Figure 12: Differential contrast image of a $Li_xV_2O_5$ particle (with respect to normalised reflectivity image when cell is at 2.2 V) during open circuit potential measurement (in a cell with electron transport layers) with illumination from a 515 nm (top) and 480 nm (bottom) LED. In the normalised reflectivity images pixels below an intensity of 0.3 are set to zero before calculation of the differential contrast image. Scale bar is 5 μm. Similar behaviour to that reported in **Figure 4** of the main text is observed with the spatial pattern in the differential contrast being independent of wavelength. There are small changes in the absolute magnitude of the differential contrast between the two wavelengths.

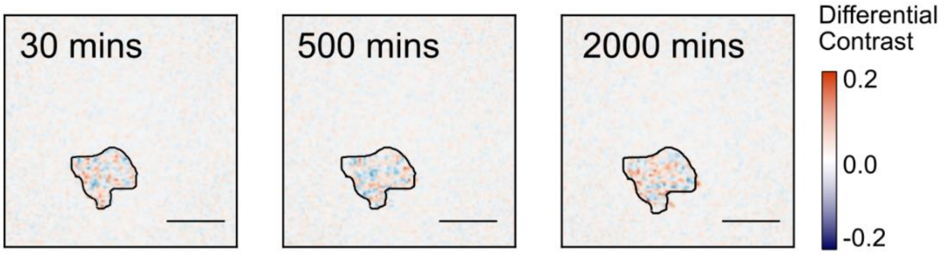

Supplementary Figure 13: Differential contrast image of a $Li_xV_2O_5$ particle (with respect to normalised reflectivity image when cell is at 2.2 V) during open circuit potential measurement (in a cell without electron transport layers) with illumination from a 515 nm LED. In this case the cell has no electron transfer layers present in it. Scale bar is 6 μm. In the normalised reflectivity images pixels below an intensity of 0.18 are set to zero before calculation of the differential contrast image. There is no change in the magnitude or spatial pattern of the reflectivity across the particle with only noise observed in the differential plots.

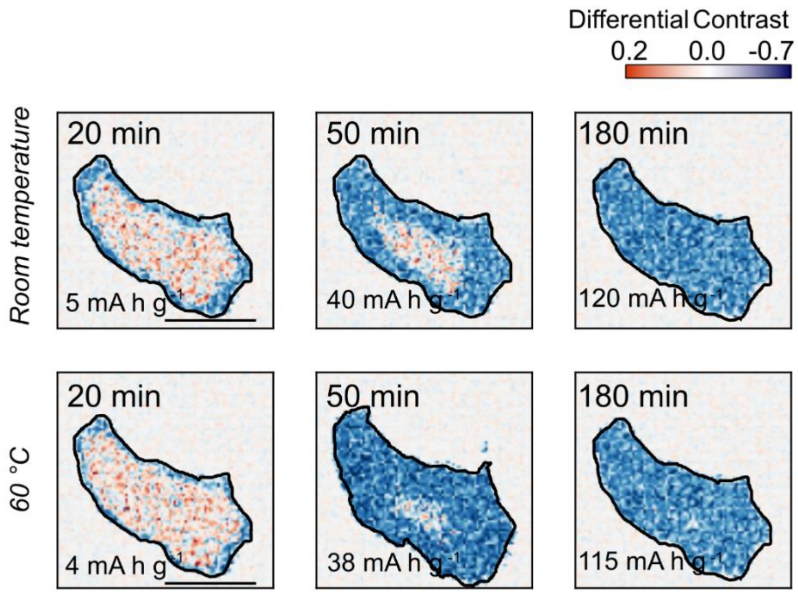

Supplementary Figure 14: Differential contrast image of a $Li_xV_2O_5$ particle (with respect to normalised reflectivity image when cell is at maximal state of charge). Experiment is performed at room temperature and whilst heating at 60 °C. Scale bar is 6 μm. In the normalised reflectivity images pixels below an intensity of 0.2 are set to zero before calculation of the differential contrast image. There is no change in the spatial pattern of the reflectivity across the particle with temperature.

Supplementary note 8: Optical imaging following photocharging and closing of the circuit with -1 nA of current

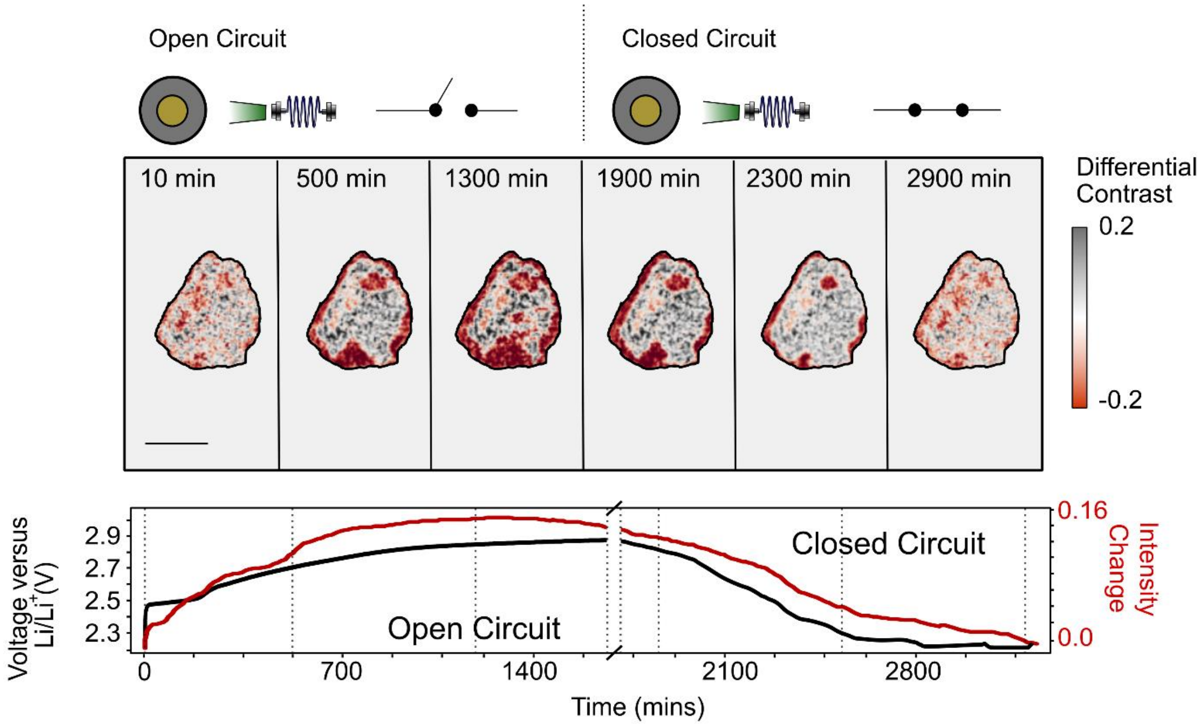

Supplementary Figure 15: Differential contrast image of $Li_xV_2O_5$ particle during open circuit potential measurement with illumination from a 515 nm LED, followed by 'closing of circuit' at ~1600 min by application of -1 nA current. In the normalised reflectivity images, pixels below an intensity of 0.27 are set to zero before calculation of the differential contrast image. In total the particle intensity in a 1 μm dimeter at the particle centre increases 16% during the photocharge, followed by a dimming by almost the same amount on closing the circuit. We note that on closing of the circuit the overall potential does not immediately drop and while between ~1600 and ~2400 mins the reflected intensity of the particle drops by ~9%, the potential remains nearly constant at ~2.8 V vs Li/Li^+. After ~2500 mins the potential then drops concomitantly with the reflected intensity. Scale bar is 3 μm.

References

(1) Ortega Arroyo, J.; Cole, D.; Kukura, P. Interferometric Scattering Microscopy and Its Combination with Single-Molecule Fluorescence Imaging. *Nat. Protoc.* **2016**, *11* (4), 617–633. https://doi.org/10.1038/nprot.2016.022.

(2) Merryweather, A. J.; Schnedermann, C.; Jacquet, Q.; Grey, C. P.; Rao, A. Operando Optical Tracking of Single-Particle Ion Dynamics in Batteries. *Nature* **2021**, *594* (7864), 522–528. https://doi.org/10.1038/S41586-021-03584-2.

(3) Xu, C.; Merryweather, A. J.; Pandurangi, S. S.; Lun, Z.; Hall, D. S.; Deshpande, V. S.; Fleck, N. A.; Schnedermann, C.; Rao, A.; Grey, C. P. Operando Visualisation of Kinetically-Induced Lithium Heterogeneities in Single-Particle Layered Ni-Rich Cathodes. *Joule* **2022**, *6*, 2535–2546. https://doi.org/10.26434/chemrxiv-2022-qb80n.

(4) Merryweather, A. J.; Jacquet, Q.; Emge, S. P.; Schnedermann, C.; Rao, A.; Grey, C. P. Operando Monitoring of Single-Particle Kinetic State-of-Charge Heterogeneities and Cracking in High-Rate Li-Ion Anodes. *Nat. Mater. 2022 2111* **2022**, *21* (11), 1306–1313. https://doi.org/10.1038/s41563-022-01324-z.

(5) Schneider, C. A.; Rasband, W. S.; Eliceiri, K. W. NIH Image to ImageJ: 25 Years of Image Analysis. *Nat. Methods* **2012**, *9* (7), 671–675. https://doi.org/10.1038/nmeth.2089.

(6) Otsu, N. Threshold Selection Method from Gray-Level Histograms. *IEEE Trans Syst Man Cybern* **1979**, *SMC-9* (1), 62–66. https://doi.org/10.1109/TSMC.1979.4310076.

(7) Pandya, R.; Valzania, L.; Dorchies, F.; Xia, F.; Mc Hugh, J.; Mathieson, A.; Tan, J. H.; Parton, T. G.; De Volder, M.; Tarascon, J.-M.; et al. Three-Dimensional Operando Optical Imaging of Single Particle and Electrolyte Heterogeneities inside Li-Ion Batteries. **2022**. arXiv [cond-mat.mtrl-sci] https://doi.org/10.48550/arXiv.2207.13073 (accessed 07/26/23).

(8) Diel, E. E.; Lichtman, J. W.; Richardson, D. S. Tutorial: Avoiding and Correcting Sample-Induced Spherical Aberration Artifacts in 3D Fluorescence Microscopy. *Nat. Protoc. 2020 159* **2020**, *15* (9), 2773–2784. https://doi.org/10.1038/s41596-020-0360-2.

(9) Gong, P.; Almasian, M.; Van Soest, G.; De Bruin, D. M.; Van Leeuwen, T. G.; Sampson, D. D.; Faber, D. J. Parametric Imaging of Attenuation by Optical Coherence Tomography: Review of Models, Methods, and Clinical Translation. *J. Biomed. Opt* **2020**, *25* (4), 40901. https://doi.org/10.1117/1.JBO.25.4.040901.

(10) Boruah, B. D.; Wen, B.; De Volder, M. Light Rechargeable Lithium-Ion Batteries Using V2O5Cathodes. *Nano Lett.* **2021**, *21* (8), 3527–3532. https://doi.org/10.1021/ACS.NANOLETT.1C00298/ASSET/IMAGES/LARGE/NL1C00298_0005.JPEG.

(11) Boruah, B. D.; Mathieson, A.; Wen, B.; Feldmann, S.; Dose, W. M.; De Volder, M. Photo-Rechargeable Zinc-Ion Batteries. *Energy Environ. Sci.* **2020**, *13* (8), 2414–2421. https://doi.org/10.1039/D0EE01392G.

(12) Datta, S.; Jo, C.; De Volder, M.; Torrente-Murciano, L. Morphological Control of Nanostructured V2O5by Deep Eutectic Solvents. *ACS Appl. Mater. Interfaces* **2020**, *12* (16), 18803–18812. https://doi.org/10.1021/ACSAMI.9B17916/ASSET/IMAGES/LARGE/AM9B17916_0005.JPEG.

(13) Hyun, H.; Jeong, K.; Hong, H.; Seo, S.; Koo, B.; Lee, D.; Choi, S.; Jo, S.; Jung, K.; Cho, H. H.; et al. Suppressing High-Current-Induced Phase Separation in Ni-Rich Layered Oxides by Electrochemically Manipulating Dynamic Lithium Distribution. *Adv. Mater.* **2021**, *33* (51), 2105337. https://doi.org/10.1002/ADMA.202105337.

(14) Brunetti, G.; Robert, D.; Bayle-Guillemaud, P.; Rouvière, J. L.; Rauch, E. F.; Martin, J. F.; Colin, J. F.; Bertin, F.; Cayron, C. Confirmation of the Domino-Cascade Model by Lifepo4/Fepo 4 Precession Electron Diffraction. *Chem. Mater.* **2011**, *23* (20), 4515–4524. https://doi.org/10.1021/CM201783Z/ASSET/IMAGES/MEDIUM/CM-2011-01783Z_0001.GIF.

(15) Wang, J.; Chen-Wiegart, Y. C. K.; Eng, C.; Shen, Q.; Wang, J. Visualization of Anisotropic-Isotropic Phase Transformation Dynamics in Battery Electrode Particles. *Nat. Commun. 2016 71* **2016**, *7* (1), 1–7. https://doi.org/10.1038/ncomms12372.
